# Myeloid related protein induces muscle derived inflammatory mediators in juvenile dermatomyositis

**DOI:** 10.1186/ar4311

**Published:** 2013-09-23

**Authors:** Kiran Nistala, Hemlata Varsani, Helmut Wittkowski, Thomas Vogl, Petra Krol, Vanita Shah, Kamel Mamchaoui, Paul A Brogan, Johannes Roth, Lucy R Wedderburn

**Affiliations:** 1 Rheumatology Unit, UCL Institute of Child Health, 30 Guilford Street, London WC1N 1EH, UK; 2 Institute of Immunology and Interdisciplinary Centre for Clinical Research IZKF, University of Muenster, Muenster, Germany; 3 Department of General Pediatrics, University Children’s Hospital Muenster, Muenster, Germany; 4 Department of Pediatrics and Adolescent Medicine, First Faculty of Medicine, Charles University in Prague and General University Hospital in Prague, Ke Karlovu 2, 12000 Prague 2, Czech Republic; 5 Thérapie des maladies du muscle strié, Institut de Myologie, UM76, UPMC Université Paris 6/U974 - Inserm/UMR7215 – CNRS, 47, bld de l’hôpital - G.H. Pitié-Salpétrière - Bâtiment Babinski, 75651 Paris, cedex 13, France

## Abstract

**Introduction:**

The aetiopathogenesis of juvenile dermatomyositis (JDM) remains poorly understood. In particular the contribution of monocytes or macrophages, which are frequently observed to be an infiltrate within muscle tissue very early in the disease process, is unknown. We hypothesised that these cells secrete the pro-inflammatory myeloid related protein (MRP) 8/14 which may then contribute to muscle pathology in JDM.

**Methods:**

In this study of 56 JDM patients, serum MRP8/14 levels were compared with clinical measures of disease activity. Muscle biopsies taken early in disease were assessed by immunohistochemistry to determine the frequency and identity of MRP-expressing cells. The effects of MRP stimulation and endoplasmic reticulum (ER) stress on muscle were tested *in vitro*. Serum or supernatant levels of cytokines were analyzed by multiplex immunoassay.

**Results:**

Serum MRP8/14 correlated with physician’s global assessment of disease activity in JDM (R = 0.65, p = 0.0003) and muscle strength/endurance, childhood myositis assessment score (CMAS, R = −0.55, p = 0.004). MRP8/14 was widely expressed by CD68+ macrophages in JDM muscle tissue. When cultured with human myoblasts, MRP8 led to the secretion of MCP-1 and IL-6, which was enhanced by ER stress. Both inflammatory mediators were detected in significantly higher levels in the serum of JDM patients compared to healthy controls.

**Conclusions:**

This study is the first to identify serum MRP8/14 as a potential biomarker for disease activity in JDM. We propose that tissue infiltrating macrophages secreting MRP8/14 may contribute to myositis, by driving the local production of cytokines directly from muscle.

## Introduction

Juvenile dermatomyositis (JDM) is a rare inflammatory disease of childhood affecting skin and muscle, frequently resulting in calcinosis, and sometimes with potentially life threatening complications including gut vasculitis and interstitial lung disease [1]. Research into the disease pathogenesis has identified abnormalities of the immune system including circulating autoantibodies targeting nuclear antigens [2], dysregulation of T helper cell subsets [3] and a prominent interferon alpha (IFNα) gene signature in the peripheral blood of JDM patients [4]. In JDM muscle, one of the earliest detectable changes is increased expression of Class I major histocompatibility complex (MHC) on skeletal muscle [5]. Class I MHC can be upregulated non-specifically in response to muscle injury [6], but in JDM is itself thought to perpetuate the disease process [7]. In animal models, forced over-expression of Class I protein leads to muscle fibre damage and a marked myeloid infiltrate into the muscle [8]. One of the mechanisms of cellular injury, known as endoplasmic reticulum (ER) stress, results from accumulation of Class I protein within the ER which then activates multiple pathways, including the unfolded protein response (UPR) culminating in muscle cell death. Gene expression profiling from patients with adult myositis, as well as animal models, confirm that the UPR is up-regulated in myositis [7,9]. Despite clear evidence that ER stress contributes to muscle cell death, it remains unclear if myocytes directly promote the inflammatory process and in particular the myeloid cell infiltrate frequently seen early in myositis, or whether this is merely a secondary response to tissue necrosis.

Once recruited, myeloid cells may potentiate the loss of muscle tissue by secreting a potent inflammatory heterodimeric protein, myeloid related protein (MRP) 8/14 (S100A8/A9) [10] which signals in a Toll-like receptor (TLR) 4 dependent manner [11] to induce apoptosis of skeletal muscle. MRP plays a pathogenic role in other childhood rheumatic diseases, including arthritis and vasculitis, and in these conditions it is a valuable biomarker of disease severity [12,13]. In JDM, the clinical and serological markers of skin and muscle disease currently available lack sensitivity [14]. In this study we examined the role of MRP8/14 as a biomarker of myositis and the mechanisms by which MRP8/14 may contribute to muscle disease. Our results show a close correlation between MRP8/14 levels in JDM serum and disease activity scores. MRP8/14 was expressed by CD68+ macrophages within JDM muscle and led to release of inflammatory mediators from muscle, an effect that was accentuated by ER stress. Our study identifies a novel pathway by which macrophage-muscle crosstalk can perpetuate inflammatory myositis.

## Methods

### Patient and control study groups

All patients were recruited to the Juvenile Dermatomyositis National Cohort Biomarker Study and Repository for Idiopathic Inflammatory Myopathies (hereafter called the JDM Cohort study), a UK-wide multicentre cohort study and biobank for JDM through the UK JDM Research Group (JDRG) [15]. The JDM cohort study had ethical approval from the North Yorkshire Multi-Centre Research Ethics Committee and was also approved by the Steering Committee of the UK JDM Cohort Study. A total of 56 patients who fulfilled the Bohan and Peter criteria for definite or probable JDM [16] were included in this study. Eighteen children (6 boys, 12 girls, mean age 5.3 years), who had a muscle biopsy taken as part of the investigation of diverse symptoms, such as clumsiness and falls, were used as controls. As previously reported [17], none of the controls received a diagnosis of myositis or myopathy and the biopsies were all reviewed by two expert histopathologists and confirmed to show normal muscle histology for age and, therefore, considered to represent suitable control samples. Ethical approval was obtained from the National Hospital for Neurology and Neurosurgery and the Institute Of Neurology Joint research ethics committee to use control muscle biopsies and serum from healthy controls. All patients (or their carers) provided consent to participate in the study.

### Laboratory and clinical measurements

Serum levels of the muscle enzymes lactate dehydrogenase (LDH) and creatine kinase (CK) were analysed by the Great Ormond Street Hospital clinical biochemistry department. Erythrocyte sedimentation rate (ESR) was measured by Great Ormond Street Hospital haematology department. Standardised outcome variables for the assessment of JDM, physician determined disease activity visual analogue score (VAS), childhood health assessment questionnaire (CHAQ), childhood myositis assessment score (CMAS) and parental global disease activity VAS [18,19] were collected prospectively as part of the UK JDM Cohort Study, as described [15].

### Measurement of MRP8/14, MCP-1 and IL-6 concentrations

Serum was extracted within three hours of collection of peripheral blood and stored at −80°C. Serum concentrations of MRP8/14 were determined by sandwich enzyme-linked immunosorbent assay (ELISA) as previously described [13]. Monocyte chemoattractant protein-1 (MCP-1) levels were measured in serum using multiplex immunoassay (Meso Scale Discovery MSD technology, Meso Scale Diagnostics, USA). MCP-1 was measured in cell culture supernatants by ELISA (eBioscience, Hatfield, UK). IL-6, and in some experiments IL-1β, IL-10, IL-17, TNF-α and IFN-γ, measurements were performed using multiplex immunoassay [20].

### Immunohistochemistry and immunofluorescence staining

Immunohistochemistry and immunofluorescence staining were performed on biopsies from quadriceps (vastus lateralis) which were snap frozen within one hour and stored at −80°C. Staining was performed on acetone fixed 7 μm cryostat sections. Immunohistochemistry was performed as described [21] using anti-human monoclonal antibodies to CD68 (KP1), CD3 (UCHT1), MHC class l heavy chain (W6/32; Novacastra, Milton Keynes, UK) and MRP8/14 (27E10 [22]). Degree of CD3 and CD68 infiltration was scored as 0, 1 or 2 as described [21]: briefly <4 cells in ×20 field scored 0, >4 cells in ×20 and/or 1 cluster scored 1, >20 cells in ×20 and/or >2 clusters in whole biopsy scored 2. For double immunofluorescence staining the following anti-human monoclonal antibodies were used: CD68 (KP1; Novocastra,), CD14 (TUK4), CD15 (C3D-1; DakoCytomation, Cambridgeshire, UK), CD163 (RM3/1 [23]) and anti-human polyclonal MRP14 [10]. Sections were incubated with primary antibodies at 4°C overnight followed by 30 minutes at room temperature with secondary antibodies sheep anti-mouse fluorescein isothiocyanate (FITC) (Sigma, Dorset, UK) and goat anti-rabbit alexa 568 (Life Technologies, Paisley, UK). Sections were mounted with Vectashield mounting medium with diamidino-2-phenylindole (DAPI) (Vector, Peterborough, UK) and analysed by fluorescent microscopy. For each patient, cells positive for lineage markers and MRP14 were counted in ten 10× fields. The mean percentage MRP14 cells expressing specific lineage markers were determined.

### Cell culture and stimulation

LHCNM2, a human skeletal myoblast cell line, was derived from the pectoralis major muscle of a 41-year-old male Caucasian heart-transplant donor, in accordance with local ethical legislation [24]. Myoblasts were grown in (Dulbecco’s) modified Eagle’s medium ((D)MEM) containing 4.5 mg/ml glucose + MEM 199 (at a ratio of 4:1), supplemented with 20% foetal calf serum (FCS), 100 IU penicillin, 100 μg streptomycin (GIBCO/Invitrogen, Paisley, UK). Myoblasts were cultured at 5 × 10^4^/ml in six-well plates at 37°C in 5% C0_2_ and the medium was refreshed when cells reached 60% confluence. To test the effects of TLR4 agonists, myoblasts were stimulated with lipopolysaccharide (LPS) or recombinant human MRP8, MRP14 or MRP8/14 [25] or left in culture medium alone. In some experiments myoblasts were pre-incubated for four hours with thapsigargin, an ER calcium-ATPase inhibitor which induces ER stress [26]. Thapsigargin was used at 0.05 μM, LPS at 100 ng/ml and MRP proteins at 5 μg/ml. Supernatants and cells were harvested as a time course over a 24-hour period. Supernatants were stored at −80°C for cytokine and chemokine measurements and cells were lysed in TRizol reagent (Invitrogen) for RNA extraction.

### RNA isolation, cDNA synthesis and PCR

RNA from both myoblast cells and muscle tissue was isolated with TRIzol according to the manufacturer’s instructions. cDNA was synthesised using superscript II RT and random hexamers (Invitrogen). TLR4 expression on muscle tissue was analysed using Quantitect TLR4 primers (Qiagen, Crawley, UK). As a read out of ER stress, expression of x-box binding protein (XBP)-1 splice variants were analysed by PCR using the following primers: forward CGGAAGCCAAGGGGAATGAA, reverse CCCAACAGGATATCAGACTCTGA, at an annealing temperature of 62°C for 35 cycles. Primers to detect HPRT mRNA (Qiagen) were used as a housekeeping control for all PCR experiments. PCR products were visualised by 1.5% agarose gel electrophoresis. MCP-1 mRNA levels were quantified using real time PCR, [27] and HPRT gene expression was used for normalisation. cDNA was amplified using SYBR green (Bio-Rad Hertfordshire, UK) and thermo cycler rotor-gene 6000 Corbert (Qiagen). Data were analysed using Rotor-gene 6000 software (Qiagen).

### Statistical analysis

Serum MRP levels, muscle enzymes and ESR were compared with clinical measures of disease activity using Spearman’s Rank. Where parametrically distributed, data were compared using 1 way or 2 way analysis of variance (ANOVA) and if appropriate paired t-tests. For non-parametric results, unpaired data were compared using the Mann–Whitney test. Differences in CD3/CD68 frequency were analysed by the Chi-Square test for trend. Results of *P* <0.05 were considered statistically significant. Analyses were carried out in SPSS version 18 (IBM, New York, USA).

## Results

### Demographics

The JDM patients in this study were representative of the whole JDM Cohort Study [15] and had evidence of moderately severe disease activity at the time of sampling with a median CMAS 33, physician’s global assessment 4.2, and CHAQ 1.3 and were analysed early in the course of their disease (median disease duration six months, Table 1 and Additional file 1: Table S1).

**Table 1:** Summary of patient demographics

**JDM patients**	**Median (inter-quartile range)**
Age at disease onset	6.0 (3.8 to 9.9) years
Number of male: female patients	14:42
Disease duration	0.5 years (0.25 to 1.8)
CHAQ	1.3 (1.0 to 2.2)
CMAS	33 (14 to 48)
Physician’s Global Assessment	4.2 (2.4 to 7.4)
LDH	1018 (781 to 1759)
CK	223 (56 to 1165)
ESR	19 (13 to 37)
Patients receiving methotrexate	14/48 (29%, 8 missing data)
Patients receiving cortico-steroids	24/51 (47%, 5 missing data)

CK, creatine kinase; CHAQ, childhood health assessment questionnaire; CMAS, childhood myositis assessment score; ESR, erythrocyte sedimentation rate; LDH, lactate dehydrogenase.

### Disease activity measures in JDM correlate with serum levels of MRP8/14

**Figure 1:**
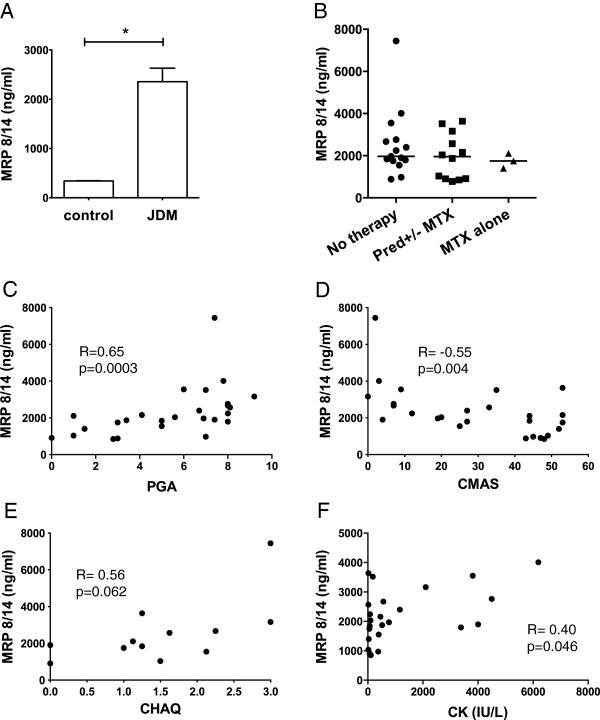
**Disease activity measures in juvenile dermatomyositis (JDM) correlate with serum MRP8/14 levels. (A)** Mean MRP8/14 concentrations were measured by ELISA in serum from 32 JDM patients and 32 paediatric controls, * *P* <0.05. **(B)** Serum MRP8/14 concentrations in JDM patients according to treatment status, n = 15 untreated, n = 12 on prednisolone+/−methotrexate, n = 3 methotrexate alone. **(C)** Correlation of serum MRP8/14 with physician global assessment (PGA), n = 26, **(D)** childhood myositis assessment score (CMAS), n = 26, **(E)** childhood health assessment questionnaire (CHAQ), n = 12 and **(F)** creatine kinase (CK), n = 25. Spearman’s Rank was used to assess correlations between variables.

Serum levels of the heterodimer MRP8/14 were significantly higher in JDM patients compared to age-matched controls but did not vary according to drug treatment (Figure 1A, B). Importantly, MRP8/14 levels strongly correlated with validated clinical measures of disease activity (Figure 1B-E), including physician global assessment of disease activity (PGA, R = 0.65, *P* = 0.0003) and strength/stamina (CMAS, R = −0.55, *P* = 0.004), and only to a limited extent with disability assessment (CHAQ, R = 0.56, *P* = 0.062) and the muscle enzyme, CK (R = 0.4, *P* = 0.046).

### Identification of MRP8/14 expressing cells in inflamed muscle

**Figure 2:**
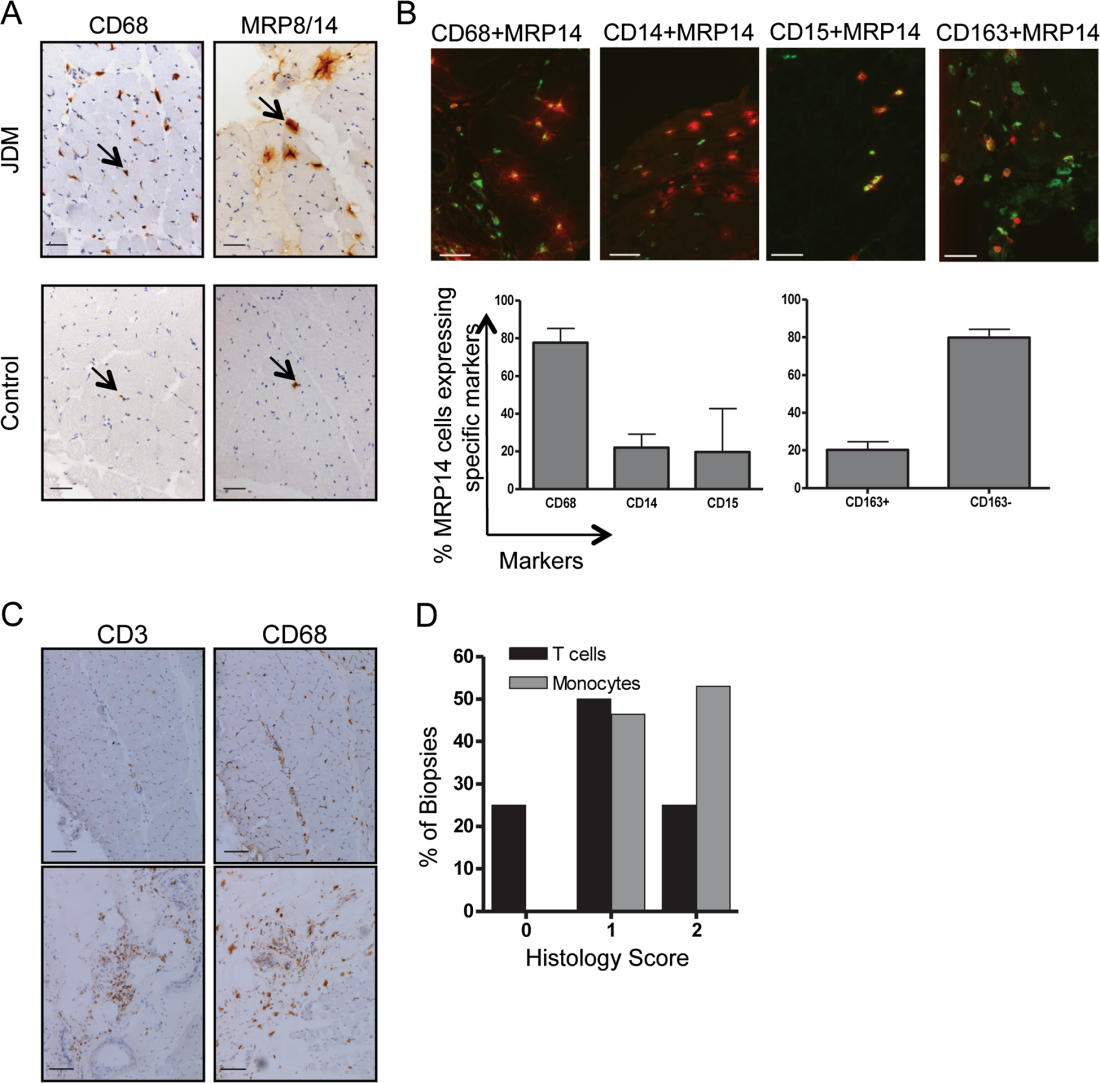
**CD68 positive myeloid cells in muscle tissue from JDM patients express MRP14 and MRP8/14. (A)** Immunohistochemical staining for CD68 (left) and MRP 8/14 (right) on muscle tissue from JDM patient (top panel) and controls (bottom panel). Positive cells stain brown as indicated by arrows. Bar represents 50 microns. **(B)** Two-colour immunofluorescence staining for infiltrating cells, lineage markers (green), MRP14 (red), double positive cells show as yellow. Bar represents 50 microns. Bar graph illustrates %MRP14+ cells expressing specific lineage markers, based on counting 10 fields at x10 magnification for each marker, bars and lines represent median and IQR respectively. **(C)** Immunohistochemical staining for CD3 and CD68 from JDM patients with a predominant macrophage infiltration, histological score of 0 for T cells and 2 for monocytes (upper panel), and mixed T cell-macrophage infiltrate, scores 2 for T-cells and 2 for macrophage (lower panel). Bar represents 100 microns. **(D)** Percentage of patients with histology scores 0, 1 and 2 for CD68 (macrophage) and CD3 (T cell) frequency in JDM muscle biopsies (n = 28, Chi square test for trend, *P* < 0.001). JDM, juvenile dermatomyositis; MRP, myeloid-related protein.

To test if infiltrating myeloid cells in myositis secrete MRP proteins, biopsies from 46 JDM patients were stained for MRP14 or the MRP8/14 heterodimer and compared to muscle biopsies from 14 age-matched controls. In JDM muscle the staining patterns observed suggested that these cells secrete MRP8/14 (Figure 2A, top right panel). The staining pattern of MRP8/14 in control samples in contrast was quite distinct (Figure 2A, bottom panel). Thus, control biopsies showed only very occasional myeloid cells, which although they stained positive for MRP8/14, showed no MRP8/14 protein secretion around the cells. To characterise MRP8/14-secreting cells in JDM biopsies, cells were analysed by dual colour immunofluorescence using polyclonal rabbit antibodies against MRP14 co-stained together with markers specific for cells of the myeloid lineage CD14, CD15 and CD68 as well as the scavenger receptor CD163 (Figure 2B). By immunohistochemistry, antibody to MRP14 reliably identified the same cells as those identified by antibody to MRP8/14 heterodimer (data not shown). The majority of MRP14 was secreted by CD68 positive cells (Figure 2B, left hand bar graph). The expression of the scavenger receptor CD163 on tissue macrophages has been described as a marker of anti-inflammatory or ‘alternately activated’ macrophages [28] and the proportion of CD163+ macrophages in muscle has been shown to increase after exercise [29]. Double staining in the JDM biopsies with MRP14 and CD163 showed that the majority of MRP8/14 secreting myeloid cells within the inflamed muscle were CD163- (Figure 2B right hand bar graph).

Examining muscle biopsies for myeloid sub-populations, we frequently observed a heavy infiltration of macrophages, often without a T cell aggregate (Figure 2C, top panels). In contrast biopsies with large numbers of T cells invariably had a significant macrophage infiltrate (Figure 2C, bottom panels). This observation raised the possibility that T cell recruitment could depend on the early macrophage infiltrate, which establishes the appropriate chemokine milieu to attract lymphocytes from the circulation. To quantify the relative abundance of macrophages and T cells in JDM muscle, we used our biopsy score tool [21] which includes a semi quantitative assessment of cell infiltration (macrophage infiltration and T cell infiltration, each scored as 0, 1, or 2). Analysis of these biopsies (n = 28) showed that muscle sections scored significantly higher for macrophage infiltration, than for T cells (*P* <0.001, Chi square test for trend, Figure 2D); indeed, 25% of these early biopsies scored 0 for T cell infiltrate. Interestingly, none of the biopsies assessed had T cell infiltration in the absence of a macrophage population, which suggests that myeloid cells may be more important in the early tissue inflammation of JDM than previously recognised.

### MRP8 drives the production of MCP-1 and IL-6 from inflamed muscle

**Figure 3:**
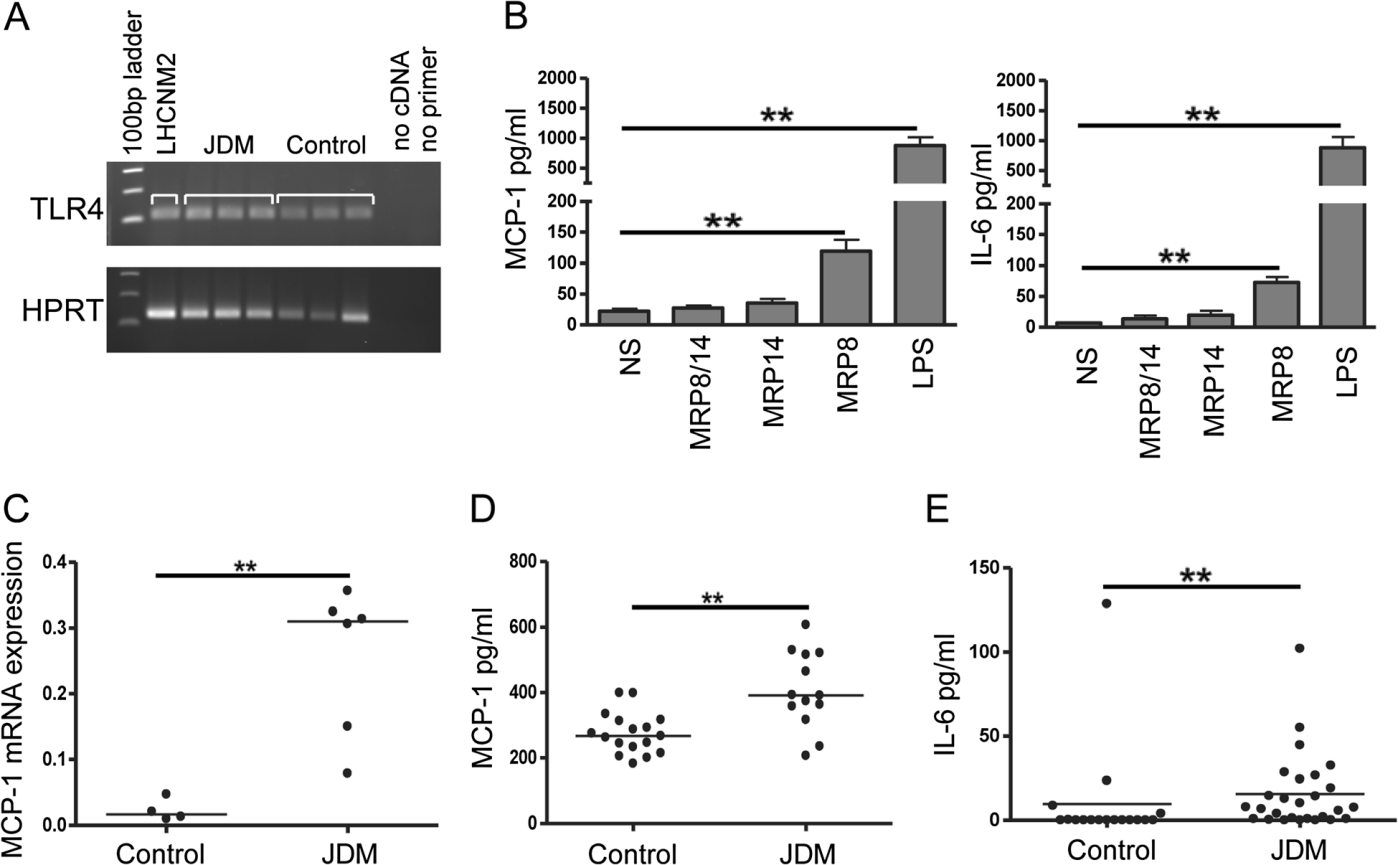
**MRP8 drives production of the chemokine MCP-1 from human skeletal muscle. (A)** Expression of TLR4 mRNA (upper panel) by RT-PCR in human skeletal myoblasts (LHCNM2) and muscle tissue from JDM and controls. HPRT mRNA was amplified as a house keeping gene (lower panel). **(B)** MCP-1 and IL-6 production by LHCNM2 cells stimulated for 24 hours with 5 μg/ml MRP8, MRP14, MRP8/14 or 100 ng/ml LPS or cultured in medium alone (NS, non stimulated); MCP-1 and IL-6 were measured in cell culture supernatants by ELISA and multiplex immunoassay, respectively; n = 6, bars and lines represent mean and SEM, respectively. Paired t -test was used for comparisons. **(C)** Quantitative PCR analysis of MCP-1 expression (relative units) in quadriceps tissue from JDM patients (n = 6) compared to age matched controls (n = 4) normalised to HPRT expression. **(D)** MCP-1 protein concentration in serum from 14 JDM patients compared to 16 aged matched controls, measured by multiplex immunoassay. **(E)** Serum IL-6 in JDM patients (n = 27) and controls (n = 16), measured by multiplex immunoassay. Lines represent medians. Mann Whitney test was used for unpaired comparisons, *P <0.05, **P <0.001. JDM, juvenile dermatomyositis; LPS, lipopolysaccharide; MCP-1, monocyte chemoattractant protein-1; SEM, standard error of the mean; TLR4, Toll-like receptor 4.

Myoblasts are known to respond to pathogen associated molecular patterns (PAMPs), including bacterial LPS, by secreting inflammatory cytokines and chemokines [30]. Since MRP8 stimulates TLR4, similar to LPS, we hypothesised that tissue macrophages secreting MRP8/14 may propagate leukocyte recruitment in JDM by inducing skeletal muscle-derived cytokines. To test this hypothesis we first confirmed expression of TLR4 on human skeletal muscle from patients, controls and cultured human myoblasts (Figure 3A). The effects of MRP homo- and heterodimers on skeletal muscle were tested by culturing LHCNM2 cells *in vitro* in the presence of LPS or MRP proteins. LPS induced high levels of IL-6 and the chemokine MCP-1 from muscle cells, whilst MRP8 led to more modest but still significant increases compared to unstimulated controls (Figure 3B). IL-1β, IL-10, IL-17, TNF-α and IFN-γ were not detected in muscle supernatants after stimulation. We have previously shown that MRP8 is the active component in TLR4 ligation [11]: again in this muscle system, MRP14 and MRP8/14 did not significantly upregulate MCP-1 or IL-6 from myoblasts. As MRP8 and MRP14 are present at increased levels in the muscles of JDM patients (Figure 2A), we asked if this would lead to a corresponding increase in tissue levels of MCP-1. Examining JDM muscle, we found significantly higher mRNA expression of MCP-1 when compared to control muscle biopsies (0.3 versus 0.02 relative units, *P* = 0.0095, Figure 3C). Analysis of JDM serum also showed significantly higher levels of MCP-1, as well as IL-6 (Figure 3D and E, respectively), than samples taken from healthy controls.

### XBP-1 and MRP8 interplay in muscle inflammation

**Figure 4:**
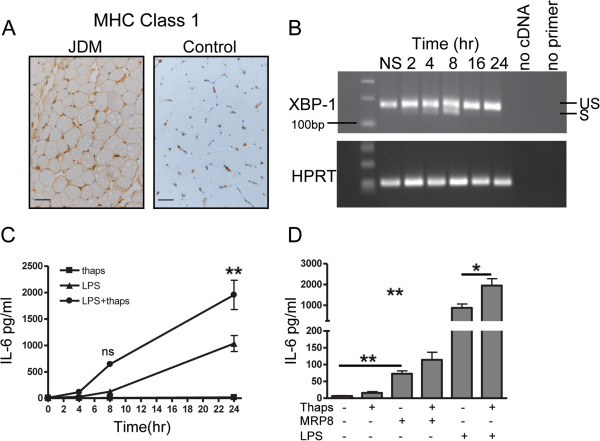
**Effect of MRP8 stimulation in combination with ER stress on IL-6 production by muscle cells. (A)** MHC class 1 expression on muscle tissue from JDM patient and age- matched control. Bar represents 50 microns. **(B)** Human skeletal muscle cells LHCNM2 cultured for 2 to 24 hours in 0.05 μM thapsigargin (Thaps), mRNA expression of XBP-1 was determined at indicated times; HPRT mRNA was measured as a control. RT-PCR products were analysed on 1.5% agarose gel and stained with ethidium bromide to visualise spliced (S), 142 bp and un-spliced (US), 168 bp XBP-1 transcripts. **(C)** Mean IL-6 production by LHCNM2 myoblasts following culture in thapsigargin, stimulated with LPS alone or with LPS following four hours pre-incubation with thapsigargin. Bars represent SEM, data analysed using two-way ANOVA. **(D)** IL-6 concentration in supernatants from LHCNM2 cells cultured with or without thapsigargin, stimulated with MRP8 or LPS alone, or MRP8 and LPS after four hours pre-incubation with thapsigargin. Bars and lines represent mean and SEM. Data analysed by 1 way ANOVA and paired *t* test *P <0.05, **P <0.001. ANOVA, analysis of variance; JDM, juvenile dermatomyositis; LPS, lipopolysaccharide; MRP, myeloid related protein; SEM, standard error of the mean; XBP-1, x-box binding protein 1.

Class I MHC over-expression in skeletal muscle (Figure 4A), which is an early pathological change in JDM, is known to be associated with ER stress, an established mechanism in the pathogenesis of myositis [7,9,31]. Recent reports have suggested that XBP-1, a key transcription factor involved in ER stress [32], may act via an alternative pathway in monocytes and augment the secretion of TLR induced cytokines [33]. It is, therefore, important to understand how the interplay between ER stress and TLR stimulation, in the form of MRP8, would influence the secretion of inflammatory mediators from skeletal muscle. LHCNM2 cells were treated with thapsigargin, to induce a stress response which was confirmed by detecting expression of the active XBP-1 splice product (26 bp smaller), seen maximally between four to eight hours (Figure 4B). As previously shown, XBP-1 splicing is regulated by a negative feedback loop [34], and 16 hours after exposure to thapsigargin, only the unspliced variant was detected. To test whether TLR ligation in muscle cells synergised with ER stress, LHCNM2 cells were cultured *in vitro* in the presence of the TLR4 agonist LPS, either alone or after pre-incubation with thapsigargin. ER stress clearly synergised with LPS to significantly increase IL-6 secretion from myoblasts when compared to stimulation with LPS alone (Figure 4C). MRP8, although acting in a TLR4 dependent manner, is less potent than LPS in activating downstream signalling cascades, including NF-κB [11]. Consequently, stimulation with MRP8 resulted in lower IL-6 levels when compared to LPS (Figure 4D), but there was still a trend for ER stress to augment the action of MRP8 (mean IL-6 114.2 pg/ml) when compared to MRP8 alone (72.8 pg/ml, *P* = 0.06). ER stress did not upregulate MCP-1 production following stimulation with either LPS or MRP8 (data not shown).

## Discussion

The assessment of disease activity in JDM, and the distinction of disease flare from deconditioning or muscle atrophy, is still largely dependent on clinical evaluation. In this study, MRP8/14 has been identified as a novel biomarker of disease activity in JDM, and found to be superior to existing serological correlates of disease. By exploring the effects of MRP8 and MRP14 on skeletal muscle *in vitro* and correlating results with *ex vivo* JDM samples, we have identified a pro-inflammatory role of MRP8 for myositic muscle that we propose contributes to the enrichment of key inflammatory mediators MCP-1 and IL-6, seen in JDM muscle and serum, which then contribute to further recruitment of inflammatory cells to muscle. MRP8 has been shown to be the active component stimulating TLR4 in murine models of inflammation whereas MRP14 seems to have a regulatory function in the MRP8/14 complex. The exact mechanisms activating the MRP8/14 heterodimer *in vivo* are currently not clear but co-stimulation may be required [35].

MRP8/14 has been shown to be a highly sensitive marker of disease activity in a range of rheumatic disorders, including arthritis, vasculitis and autoinflammatory disease [12,13]. As a biomarker, MRP8/14 has many characteristics that make it suitable for both clinical and research use; it is easily detected in serum even at low levels, is already in clinical use to detect gut inflammation [36] and is stable in clinical serum samples even when transported at room temperature. Most recently, we have successfully used MRP8/14 to identify patients with juvenile arthritis who are likely to remain in remission following withdrawal of immunosuppression by methotrexate [37]. This proof of concept study confirms a role for MRP8/14 in the detection of sub-clinical disease activity in rheumatic disorders and could potentially apply to JDM to assist with the withdrawal of immunosuppression.

Other biomarkers have been identified in JDM, including the IFNα gene signature and IFN induced chemokines [4,38-40]. IFNα is produced by plasmacytoid dendritic cells (pDC), and induces transcriptional activators which bind downstream response elements in promoter sequences (IFN-stimulated response elements, ISRE), enhancing the transcription of many immune related genes including the chemokine MCP-1 [41]. Our results suggest that MCP-1 can also be produced by a MRP-dependent pathway, by muscle fibres themselves in JDM. It is, therefore, of interest, that in a recent report MCP-1, an IFNα associated chemokine correlated better with disease activity than the IFNα gene signature itself [4]. This may suggest that IFNα-independent production of MCP-1, including muscle derived MCP-1, may play a role in juvenile myositis.

It is striking that JDM biopsies that were taken relatively early in disease (median disease duration six months), already show a major infiltration of monocytes/macrophages, often in the absence of cells from the adaptive immune system. Previous studies have not clearly defined the role of such infiltrating myeloid cells in muscle cell damage and inflammation during myositis. Our results clearly show that MRP8 and MRP14 are secreted by CD68+ infiltrating cells and that these are largely CD163- suggesting that they are indeed pro-inflammatory in phenotype. We propose that the local secretion of MRP proteins by these cells has several downstream effects, including the stimulation of muscle to produce MCP-1 and IL-6. MCP-1 secreted by skeletal muscle, in response to MRP, may then play an important role in propagating the inflammatory infiltrate. Cells recruited into DM muscle, including monocytes and memory T cells, express high levels of CCR2, the sole receptor for MCP-1 [42,43]. MRP8 and MRP14 may be important in linking an initial innate immune response with a later adaptive one by recruiting CCR2+ memory T cells and supporting the differentiation of local B cells into plasma cells as this is dependent on signalling through CCR2 [44]. As further evidence of the importance of MCP-1 in myositis, animal models have implicated this chemokine in a transgenic model of selective over expression of self MHC Class l in skeletal muscle, MHC over expression induced MCP-1 production [8] and, in a model of viral induced myositis, blockade of MCP-1 significantly attenuated muscle inflammation [45].

Our results demonstrating that MRP8 induced IL-6 and MCP-1 secretion by myoblasts add to a growing body of evidence that suggests that muscle itself contributes to the inflammatory process [8,46]. To test how muscle derived cytokine secretion would be altered by non-immune insults to the muscle, known to occur in DM [7-9], we adopted a thapsigargin induced model of muscle ER stress. Using this system we have now identified ER stress as a mechanism for priming myoblasts to secrete IL-6 in response to a second signal, such as macrophage derived MRP8 binding TLR4. This is pertinent to myositis as IL-6 is known to correlate with disease activity in idiopathic inflammatory myositis (IIM) and IL-6 blockade attenuates muscle inflammation in some mouse models [47]. Given that IL-6 is produced by a range of immune cells, including macrophages, B and T cells, it is difficult to discern the exact contribution made by skeletal muscle towards the enrichment of this cytokine in JDM serum. Nevertheless, MCP-1 and IL-6 appear to be tightly co-regulated in JDM serum [4] and, given that both are expressed by inflamed muscle, it is possible that the close correlation between serum levels and muscle disease activity [4,40] is explained by the muscle itself being a key source of these cytokines in JDM.

One limitation of our study is that we are unable to exclude the action of MRP8 on other receptors apart from TLR4, such as the for advanced glycation end products (RAGE) [48]. However, data from the murine system would suggest that TLR4 is the dominant receptor for MRP8 [35].

This study contributes novel insights into the possible roles of macrophage derived MRP8 and MRP14 in driving production of chemokines and cytokines by muscle cells. These data emphasise the importance of skeletal muscle as an organ with the potential for immune functions and demonstrate how cross talk between muscle and the innate immune system can be instrumental in sustaining further adaptive responses as well as on-going inflammation in autoimmune muscle disease.

## Conclusions

Our results highlight the relationship between serum MRP8/14 levels and disease activity in JDM, and identify a novel mechanism by which macrophage derived MRP8/14 may directly activate muscle cells and, thereby, perpetuate inflammatory myositis. Further prospective studies are required to test the role of MRP8/14 as a putative biomarker for disease activity in JDM.

## Abbreviations

ANOVA: Analysis of variance; bp: Base pair; CHAQ: Childhood health assessment questionnaire; CK: Creatine kinase; CMAS: Childhood myositis assessment score; ELISA: Enzyme-linked immunosorbent assay; ER: Endoplasmic reticulum; ESR: Erythrocyte sedimentation rate; IFNα: Interferon alpha; JDM: Juvenile dermatomyositis; LDH: Lactate dehydrogenase; LPS: Lipopolysaccharide; MCP-1: Monocyte chemoattractant protein-1; MHC: Major histocompatibility complex; MRP: Myeloid related protein; PCR: Polymerase chain reaction; TLR: Toll-like receptor; UPR: Unfolded protein response; VAS: Visual analogue score; xbp: x-box binding protein.

## Competing interests

The authors declare that they have no competing interests.

## Authors’ contributions

HV carried out immunohistochemistry, myoblast culture and PCR. HW, TV and JR assayed MRP and provided technical support. VS, PK and PB assayed MCP-1. KM provided the myoblast cell line. KN, KM and HV analysed data and performed statistical analyses. HV, KN, KM, JR and LW conceived of the study, designed experiments and participated in its design and coordination and helped to draft the manuscript. All authors read and approved the final manuscript.

## Supplementary Material

Additional file 1 Patient demographics and clinical scores. Click here for file
